# Dual Antiplatelet Therapy Duration in Acute Coronary Syndrome Patients: The State of the Art and Open Issues

**DOI:** 10.1155/2020/6495036

**Published:** 2020-04-07

**Authors:** Monica Verdoia, Cyril Camaro, Elvin Kedhi, Marco Marcolongo, Harry Suryapranata, Giuseppe De Luca

**Affiliations:** ^1^Division of Cardiology, Ospedale degli Infermi, ASL Biella, Biella, Italy; ^2^Division of Cardiology, Azienda Ospedaliera-Universitaria “Maggiore della Carità”, Eastern Piedmont University, Novara, Italy; ^3^Department of Cardiology, UMC St Radboud, Nijmegen, Netherlands; ^4^Division of Cardiology, AZ Sint Jan, Bruges, Belgium

## Abstract

Conflicting results have been reported so far in pooled analyses and studies evaluating the optimum duration of dual antiplatelet therapy (DAPT) in acute coronary syndrome (ACS) patients. However, randomized clinical trials dedicated to this specific setting of higher thrombotic risk patients have only recently been completed, pointing at the noninferiority of a shorter strategy as compared to the traditional 12-month DAPT, furthermore allowing to reduce the risk of major bleeding complications. Therefore, a reconsideration of current clinical practice and guidelines should be certainly be advocated in light of the most recent updates, especially among ACS patients treated with percutaneous coronary intervention (PCI) and modern drug-eluting stents (DES). Our aim was to provide a comprehensive review of the available evidence on the optimal DAPT duration in ACS patients.

## 1. Background

Dual antiplatelet therapy (DAPT) using a combination of aspirin and a P2Y12 inhibitor (either a thienopyridine—clopidogrel or prasugrel—or ticagrelor) has represented the key point in the achievement of the significant prognostic improvements observed among patients with acute coronary syndrome (ACS) [1–3].

The European Society of Cardiology (ESC) and the American College of Cardiology (ACC)/American Heart Association (AHA) recommend 12 months of DAPT after an acute cardiovascular event irrespective of the revascularization strategy, in both patients managed medically and those undergoing percutaneous coronary interventions [4, 5]. However, the latter certainly represents a higher thrombotic risk subgroup, where DAPT is mandatory in the first months after stent implantation until re-endothelization is complete, in order to prevent the device thrombosis and restenosis [6, 7].

Indeed, the recent achievements in stent technologies, with thinner struts, absent or bioresorbable polymer, and sharper imaging-assisted implantation techniques, have further improved clinical outcome with drug-eluting stents (DES), with reduced rates of restenosis and thrombotic complications, allowing a shorter DAPT duration (even as short as 1 month) [8–12].

However, whilst a progressive shortening of DAPT, driven by the DES technology is actually pursued in clinical practice, allowing to lower the rate of major bleeding complications, on the contrary, an opposite tendency towards a prolongation of the therapy has demonstrated additional anti-ischemic benefits in those higher risk patients, as those presenting with an ACS. The several trials and meta-analyses performed so far have failed to provide consistent indications on the optimal duration of DAPT in ACS [12–14], potentially due to the modest amount of data specifically addressing this population and in consideration of the use of the one-size-fits-all approach, not accounting for the significant heterogeneity of the bleeding and thrombotic risk in these patients.

The recent results of large-scale randomized trials [15, 16] evaluating the prognostic impact of a shorter vs. traditional 12-month DAPT in ACS patients undergoing coronary stenting with newer DES allow to reconsider the current therapeutic strategies in terms of antiplatelet treatment.

## 2. Dual Antiplatelet Therapy (DAPT) Duration in ACS: The Standard of 12 months

For many years, the administration of DAPT for a 12-month period after ACS has been indicated, primarily based on the CURE [17] trial, a study that was conducted 20 years ago and enclosing only a minority of patients treated with an early invasive revascularization strategy and even less receiving stent implantation. However, in that trial, the largest proportion of benefits among patients randomized to 1-year DAPT were observed before angiography in light of the earlier administration of clopidogrel as compared with those randomized to 1 month, while the difference between the two study groups became nonsignificant after 90 days after PCI.

In addition, the prolongation of DAPT has progressively required to deal with a higher occurrence of hemorrhagic events, being associated with impaired survival and enhanced ischemic complications.

In fact, in a substudy of the Acute Catheterization and Urgent Intervention Triage strategY (ACUITY) and Harmonizing Outcomes with RevasculariZatiON and Stents in Acute Myocardial Infarction (HORIZON-AMI) trials [18], including over 16,000 patients, the authors observed a strong independent relation of low hemoglobin levels and mortality, and similarly in the Paris registry [19], anemia emerged as the most important predictor of early DAPT discontinuation and recurrence of major cardiovascular events.

In addition, bleeding events certainly play an even greater role with more potent antiplatelet agents such as ticagrelor and prasugrel, resulting superior to clopidogrel in terms of platelet inhibition and antithrombotic protection in the respective PLATO [20] and TRITON-TIMI 38 [21] trials, being therefore currently indicated as a first-line strategy (class IA) over clopidogrel in the settings of ACS. However, higher percentages of non-CABG-related TIMI major bleeding events were observed in both PLATO (2.9 vs. 2.2%; *p*=0.003) and TRITON-TIMI 38 (2.4 vs. 1.8%; *p*=0.003) studies as compared with clopidogrel.

However, the efforts in reducing the duration of DAPT have been, so far, prevented by the fear of late (>30 days) and very late (>1 year) stent thrombosis, events that have prevented the spread of DES for many years. The development of newer generations of DES, with thinner struts, more predictable drug release, and lower grade of inflammation, thanks to biodegradable polymer or polymer-free technology, have allowed a faster re-endothelization, therefore reducing the rate of thrombotic complications to neglectable levels (<1%) and offering promising outcomes even with a shorter 1 to 6 months DAPT [12–16, 22].

Since 2012, after the publication of the PRODYGY trial [23], that randomized 2013 patients to 6 vs. 12 months of DAPT after PCI, further 14 trials have addressed the feasibility of reducing the period of DAPT [15, 16, 24–35], documenting the noninferiority of a shorter strategy as compared with the traditional 12 months in terms of anti-ischemic protection.

However, the limitations of the available studies have prevented, so far, the inclusion of these findings in routine clinical practice, in particular, the heterogeneity of the enrolled population and stent strategy, the nonuniformity of the primary endpoint, and the low ischemic events rate, conditioning the reduced power of these trials.

In the most recent guidelines, the optimal duration of DAPT has been lowered to at least 6 months only in stable patients although shorter or longer strategies could be reasonably considered according to patients' risk profile, requiring to balance between the thrombotic and hemorrhagic risk [4].

On the contrary, the traditional period of 12-month DAPT is still recommended in ACS patients, not accounting for the results of the recent studies, due to the modest amount of available literature. In fact, in previous trials, patients presenting for an acute event represented only 20–30% of the study population, as underlined in Table 1. However, it is also indicated that “in specific clinical scenarios, this standard DAPT duration can be shortened (<12 months) or extended (>12 months).” Nevertheless, the exact definition for these scenarios is still unclear.

In the multicenter DAPT STEMI trial [15], a total of 870 STEMI patients treated with primary angioplasty and RESOLUTE Onyx Stent who were taking DAPT and were event-free at six months were randomized 1 : 1 to single antiplatelet therapy or to DAPT for an additional six months. New ADP antagonists were similarly used in both groups (58%). All patients who were randomized were then followed for another 18 months (i.e., 24 months after the primary PCI). The primary endpoint (composite of all-cause mortality, any MI, any revascularization, stroke, and TIMI major bleeding at 18 months after randomization) occurred in 4.8% of patients receiving single antiplatelet therapy vs. 6.6% of patients receiving DAPT (*p*_non-inferiority_ = 0.004). In the multicenter SMART DATE trial [24], a total of 2712 ACS patients treated with PCI and DES with permanent (Xience or RESOLUTE Onyx) or bioresorbable (ORSIRO) polymer were randomly assigned to 6-month DAPT (*n* = 1357) and 12-month or longer DAPT (*n* = 1355). Clopidogrel was used in 79.7% of patients in the 6-month DAPT and in 81.8% of patients in the 12-month DAPT. The primary endpoint (composite of all-cause death, MI, or stroke at 18 months) occurred in 4.7% with the 6-month DAPT and in 4.2% with the 12-month DAPT (*p*_non-inferiority_ = 0.03). Although all-cause mortality did not differ significantly between 6-month DAPT and 12-month DAPT (2.6% vs. 2.9%, *p* = 0.90) and neither did stroke (0.8% vs. 0.9%, *p* = 0.84) and ST (1.1% vs. 0.7%, *p* = 0.32), MI occurred more frequently in the 6-month DAPT than in the 12-month DAPT group (1.8% vs. 0.8%; *p* = 0.02). No significant difference was observed in the rate of BARC type 2–5 bleeding between the two groups (2.7% vs. 3.9%; *p* = 0.09).

The REDUCE trial [16] compared a very short DAPT (3 months) vs. a standard 12-month DAPT strategy in a total of 1496 ACS patients successfully treated with a new-generation DES (COMBO), including almost 50% STEMI patients and large use of new ADP antagonists (almost 60%). Differently from the DAPT STEMI but similarly to the SMART DATE, patients were randomized during initial hospitalization.

In this study, a 3-month DAPT strategy was not inferior to 12-month DAPT with regards to the primary endpoint (composite of mortality, MI, ST, stroke, TVR, or bleeding (BARC II, III, and V)). Similar outcome between the two groups was observed at 2-year follow-up (11.6% vs. 12.1%, respectively) and also confirmed in the per-protocol analysis, actual treatment analysis, and for major subgroups such as age, diabetic status, gender, type of ACS (STEMI vs. NSTEMI/ACS), and kidney function.

No significant differences were observed in the secondary endpoints (mortality, MI, ST, TVR, and bleedings) although cardiac mortality and stent thrombosis were numerically higher in the three-month DAPT group.

**Figure 1:**
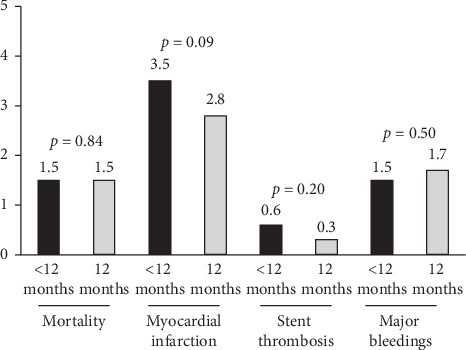
Bar graph showing the rate of major clinical events in clinical trials comparing 12-month dual antiplatelet strategy (DAPT) vs. a shorter strategy followed by ASA alone.

In a recent comprehensive meta-analysis restricted to ACS, including 17,941 patients [12], a shorter DAPT strategy was associated with a nonsignificant reduction in bleedings, whereas no difference in cardiovascular mortality, MI, and ST was observed with a shorter vs. standard 12-month DAPT (Figure 1).

**Figure 2:**
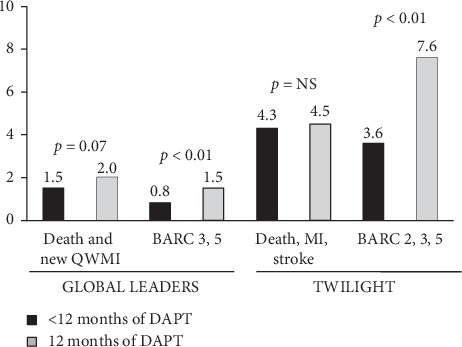
Bar graph showing the rate of major clinical events in clinical trials comparing 12-month dual antiplatelet strategy (DAPT) vs. a shorter strategy followed by ticagrelor alone.

Several trials have recently investigated a short DAPT strategy with the drop of aspirin that has been claimed as the major determinant of gastrointestinal bleeding complications. The GLOBAL LEADERS trial [36] compared 1-month DAPT followed by 23-month ticagrelor vs. standard 12-month DAPT. This trial included 130 secondary/tertiary care hospitals in different countries, with 15,991 unselected patients with stable coronary artery disease or ACS undergoing PCI. The non-prespecified, post hoc analysis restricted to ACS patients, including 7487 patients (3750 assigned to 1-month DAPT followed by ticagrelor therapy and 3737 to standard 1-year DAPT), has been recently published. Between 31 and 365 days after randomization, the primary outcome (composite of all-cause death or new Q-wave myocardial infarction) occurred in 55 patients (1.5%) in the experimental group and in 75 patients (2.0%) in the reference group (HR = 0.73; *p*=0.07); investigator-reported Bleeding Academic Research Consortium-defined bleeding type 3 or 5 occurred in 28 patients (0.8%) with 1-month DAPT and in 54 patients (1.5%) with 1-year DAPT (HR = 0.52; *p*=0.004) (Figure 2).

The TWILIGHT trial [37] included patients undergoing PCI who were at high risk for ischemic or hemorrhagic complications and who completed a 3-month course of dual antiplatelet therapy with ticagrelor plus aspirin. They were, thereafter, randomized to single antiplatelet therapy (SAPT) with ticagrelor or DAPT up to 12-month follow-up. The primary endpoint was the occurrence of BARC 2, 3, or 5, whereas the secondary endpoint was the combined occurrence of all-cause death, nonfatal MI, or stroke. Out of 7119 patients enrolled in the study, 4614 had ACS. In this population, SAPT, as compared with DAPT, was associated with a significant reduction in the primary safety endpoint (3.6% vs. 7.6%; *p* < 0.01), without any difference in the secondary endpoint (4.3% vs. 4.5%) (Figure 2).

## 3. Prolonged DAPT beyond 1 Year after ACS for Secondary Prevention

In the last few years, several trials and meta-analyses have addressed different strategies of DAPT duration, attempting to decrease either the ischemic or the hemorrhagic complications and improve the outcomes of ACS patients.

In particular, the option of prolonging the treatment with DAPT beyond 12 months has been explored in different trials [38–40], based on the observations in older studies that first-generation DES was associated with a significantly higher risk of very late ST. In the International Drug-Eluting Stent Event Registry of Thrombosis (DESERT), in fact, the majority of ST events occurred after 1 year (75%) and continued to be observed for as long as 7.3 years [41]. Similarly, the large National Heart, Lung, and Blood Institute Dynamic Registry reported a significant 4-year reduction of mortality in patients with extended DAPT after DES implantation [42].

On the contrary, in the large nonrandomized registry, the Coronary Revascularization Demonstrating Outcome (CREDO)-Kyoto Registry Cohort-2 [43], prolonged thienopyridine therapy beyond 1 year did not reduce ischemic events, but showed a trend toward increased bleeding. A similar risk was also underlined in the Dual Antiplatelet Therapy (DAPT) Study [40], an international multicenter, randomized trial that compared 30 with 12 months of dual antiplatelet therapy after PCI, where the continuation of treatment beyond 12 months was associated with a reduction in ischemic events (stent thrombosis and myocardial infarction), but also increased noncardiovascular death, potentially driven by a marked raise in hemorrhagic complications. Moreover, almost 50% of these who avoided myocardial infarctions were nonstent related, and therefore associated to the prevention of “de novo” events, with no final impact on survival, as concluded also by subsequent meta-analyses [44], thus leaving considerable uncertainty with respect to the appropriateness of the extension of DAPT beyond the recommended period except than in selected subsets of patients at very high risk of recurrent ACS.

However, none of these studies specifically addressed to the ACS population and neither conducted with newer generations of DES.

**Figure 3:**
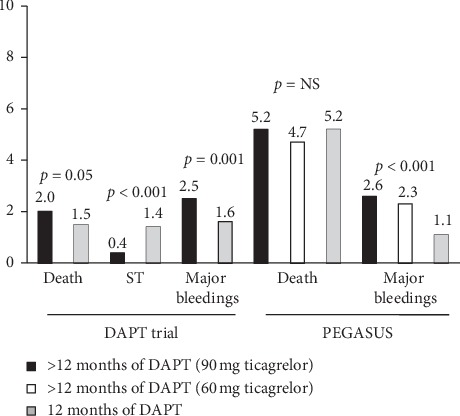
Bar graph showing the rate of major clinical events in clinical trials comparing 12-month dual antiplatelet strategy (DAPT) vs. a longer (>12 months) strategy.

In the Dual Antiplatelet Therapy (DAPT) trial [40], a total of 9961 patients undergoing DES implantation were randomly assigned at 1-year follow-up to continue thienopyridine treatment up to 30 months or to receive placebo. Prolonged DAPT was associated with reduced stent thrombosis (0.4% vs. 1.4%; *p* < 0.001) and major adverse cardiovascular and cerebrovascular events (4.3% vs. 5.9%; *p* < 0.001). The rate of myocardial infarction was lower with prolonged DAPT than with placebo (2.1% vs. 4.1%; *p* < 0.001). However, prolonged DAPT was associated with higher all-cause mortality (2.0% vs. 1.5%; *p*=0.05) and moderate or severe bleeding (2.5% vs. 1.6%; *p*=0.001), as in Figure 3. A post hoc analysis showed less benefits in thrombotic complications with prolonged DAPT in patients receiving new-generation DES (Xience) as compared with first-generation SES or PES (*p* int = 0.048). Besides, another subanalysis showed that the reduction of MACCE for prolonged DAPT was greater for MI patients (3.9% vs. 6.8%; *p* < 0.001) compared with those with no MI (4.4% vs. 5.3%; *p*=0.08; interaction *p*=0.03) [45].

In the PEGASUS trial [46], a total of 21,162 patients with a previous (1 to 3 years earlier) myocardial infarction were randomly assigned 1 : 1 : 1 fashion to ticagrelor 90 mg twice daily, ticagrelor 60 mg twice daily, or placebo and followed up for a median of 33 months. The primary efficacy end point was the composite of cardiovascular death, myocardial infarction, or stroke. The primary safety end point was thrombolysis in myocardial infarction (TIMI) major bleeding.

As displayed in Figure 3, the two ticagrelor doses each reduced, as compared with placebo, the rate of the primary efficacy end point (composite of cardiovascular death, myocardial infarction, or stroke) (*p*=0.008; *p*=0.004). Rates of TIMI major bleeding were higher with ticagrelor (2.60% with 90 mg and 2.30% with 60 mg) than with placebo (1.06%) (*p* < 0.001 for each dose vs. placebo). An overall similar all-cause mortality was observed in the three groups.

Nevertheless, more complex patients were excluded from these studies, such as those with a high bleeding risk or those with more advanced coronary disease, including patients with left main disease or multivessel CAD and incomplete revascularization.

Thus, based on the current findings, a standard 12-month DAPT cannot certainly be systematically applied to all ACS patients, whilst an individualized therapy of shortened or extended therapy according to patients' risk profile should be advocated.

In fact, the current guidelines do recommend a prolonged DAPT in patients at high risk for thrombotic complications but with low bleeding risk (class 2A).

However, further large-scale studies will allow to better define the criteria for the stratification of the patients and tailoring of DAPT.

## 4. DAPT Optimization in Special Populations: Towards and Individualized DAPT Duration

In the last decades, the complexity of patients admitted for an ACS has progressively increased. In fact, with the progressive ageing of the population, among 30% of patients with an acute ischemic event are in advanced age, displaying a higher rate of comorbidities, such as diabetes, renal failure, and a more severe coronary disease, therefore requiring extensive stenting and the management of difficult anatomies. Thus, both thrombotic and bleeding risk are enhanced among these patients, challenging the optimization of DAPT.

Several studies have attempted so far to identify the best criteria for the selection of the optimal DAPT duration. In particular, several risk scores have been developed, derived from large randomized trials, although the majority were developed for the prediction of events occurring mainly during hospital stay or soon after discharge. The newer “DAPT score” [47], derived from the 11,648 patients enrolled in the Dual Antiplatelet Therapy study (DAPT) trial, may be useful for decisions about extending DAPT in patients treated with coronary stent implantation, suggesting that a prolonged > 12-month therapy may be favorable for those with a score ≥2. Diabetes mellitus, current cigarette use, prior PCI or prior MI, congestive heart failure or left ventricular ejection fraction <30%, MI at presentation, vein graft PCI, and stent diameter <3 mm increase the thrombotic risk, while a score reduction is warranted by advanced age, especially for patients >75 years of age. On the contrary, for the prediction of bleedings, the most recent score endorsed by guidelines is represented by the PRECISE-DAPT (PREdicting bleeding Complications In patients undergoing Stent implantation and subsEquent Dual Anti-Platelet Therapy) [48], enclosing a five-item (age, CrCl, hemoglobin, white blood cell count, and prior spontaneous bleeding) prediction algorithm for out-of-hospital bleeding in patients treated with DAPT. It was observed that among patients deemed at high bleeding risk (PRECISE-DAPT score ≥ 25), prolonged DAPT was associated with no ischemic benefit but a remarkable bleeding burden leading to an NNT for harm of 38, whilst a PRECISE-DAPT score <25 was associated with a significant reduction in ischemic endpoints.

Nevertheless, the exact prognostic role of these scores in the context of real-life ACS patients, often displaying a greater complexity as compared with subjects enrolled in randomized clinical trials, is still unknown.

In addition, it should be accounted that about one-third of ACS patients nowadays displays an indication to oral anticoagulation [1, 49–51], increasing the hemorrhagic risk and therefore raising uncertainty not only on the timing of DAPT discontinuation but also on the optimal combination of the antithrombotic and antiplatelet agents and the selection of the drug to be dismissed.

**Figure 4:**
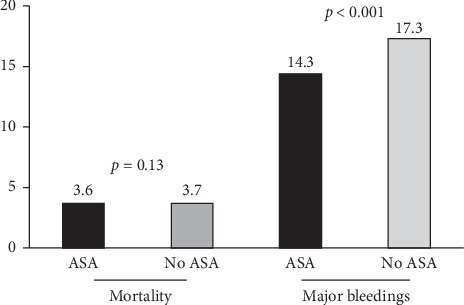
Bar graph showing the rate of major clinical events (mortality and major bleedings) with ASA-free or traditional dual antiplatelet strategy (DAPT) in patients receiving concomitant oral anticoagulation.

In fact, recently completed trials have introduced the opportunity of an aspirin-free dual therapy in post-PCI patients [36, 37, 49–53], providing comparable mortality and a reduction in major bleedings as compared with triple therapy, introducing the concept that ASA could be no more a pillar treatment in coronary disease, especially in the era of novel oral anticoagulants (Figure 4).

The WOEST trial [52] was the pioneer trial in the proposition of an aspirin-free strategy after PCI in AF patients (vitamin K antagonists and clopidogrel). In this trial including 573 patients, a dual therapy strategy was associated with a significant reduction in major bleeding complications without any excess in thrombotic complications as compared with triple therapy.

In the RE-DUAL PCI [49], patients were randomized to either dabigatran plus P2Y12 inhibitor or to a triple therapy with warfarin. However, in the warfarin arm, DAPT was continued for a maximum of three months, with a subsequent drop of ASA, in ACS patients, that represented 52% of the study population.

Similarly, the PIONEER AF-PCI [50] that enrolled about half of the patients with ACS showed a comparable efficacy, but a reduction of major bleedings, with rivaroxaban plus a P2Y12 inhibitor for 12 months as compared with warfarin plus DAPT. However, in this study, the duration of triple therapy was planned for 1, 6, or 12 months, with warfarin plus ASA being continued thereafter.

Analogous conclusions were reached also in the most recent AUGUSTUS trial [51], enclosing 37.3% of ACS patients treated with PCI and 23.9% of ACS patients medically managed that were randomized to P2Y12 plus apixaban or warfarin, with or without ASA.

The advantages of a dual therapy with NOAC and ADP antagonists, as compared with triple therapy with VKA have been confirmed with edoxaban in the recent ENTRUST-AF PCI trial [53].

However, the large heterogeneity of the strategies proposed for the management of these high-bleeding risk subjects, as described in Table 2, has not led so far to a joint agreement in terms of triple therapy, especially in ACS patients.

## 5. Future Perspectives

Real-life experience has pointed at the presence of several clinical and angiographic factors which should be acknowledged for the estimation of the balance between the thrombotic and the ischemic risk in ACS patients, therefore for the planning of the optimal duration of DAPT. Evidence from the recent studies has clearly shown that technological improvements and the broad spectrum of pharmacological adjuncts nowadays offer a wide range of combinations, allowing to tailor the antithrombotic therapy according to patients' risk profile.

Therefore, it appears that an individualized approach of shortening or prolonging DAPT according to the patients' characteristics should be recommended. Due to current evidences, it is undoubtful that a shorter DAPT strategy may be reasonable among ACS patients, potentially leaving a standard 12-month or longer therapy to high-risk patients with extensive coronary disease, complex anatomy, and percutaneous intervention, and therefore at high risk for thrombotic complications, in the presence of low risk of bleedings. Due to the results of the GLOBAL LEADERS and TWILIGHT trial [36, 37], it may be questioned whether aspirin is still needed in the treatment of ACS in the era of new ADP antagonists. Further investigations are certainly needed to solve this issue.

While many trials [49–53] have recently demonstrated that a dual therapy (NOAC and ADP antagonists) may be the preferred strategy after percutaneous intervention in patients needing chronic oral anticoagulation, further investigations are certainly warranted in ACS patients.

Future large dedicated studies will certainly help to define the criteria that should be accounted for the stratification of the patients and for the weighting of the ischemic and hemorrhagic risk that should guide an optimal tailored antiplatelet therapy.

## Figures and Tables

**Table 1:** Characteristics of randomized studies comparing different durations of dual antiplatelet therapy.

Study	Antiplatelet treatment				ACS patients (n/N)	Primary efficacy endpoint	Primary efficacy result	Primary safety endpoint	Primary safety result
	Strategy shorter DAPT	Months	Strategy longer DAPT	Months					
RESET	ASA + clopidogrel	3	ASA + clopidogrel	12	601/2117	Composite death from all-cause myocardial infarction or stent thrombosis	—	TIMI	—
ISAR-SAFE	ASA + clopidogrel	6	ASA + clopidogrel	12	1601/4000	Composite of death, myocardial infarction, stent thrombosis, stroke, or TIMI major bleedings	—	TIMI	—
DAPT STEMI	ASA + prasugrel, ticagrelor (preferred above clopidogrel)	6	ASA + prasugrel, ticagrelor (preferred above clopidogrel)	12	870/870	All-cause mortality, myocardial infarction, any revascularization, stroke, TIMI major bleeding	—	TIMI/BARC 3	—
I-LOVE-IT 2	ASA + clopidogrel	6	ASA + clopidogrel	12	1496/1829	Composite of cardiac death, target vessel myocardial infarction (TVMI), or clinically indicated target lesion revascularization (CI-TLR)	—	BARC 3–5	—
REDUCE	ASA + prasugrel, ticagrelor (preferred above clopidogrel)	3	ASA + prasugrel, ticagrelor (preferred above clopidogrel)	12	1496/1496	All-cause death, MI, ST, stroke, TVR, or bleeding	—	BARC 2–5	—
SMART DATE	ASA + any P2Y12 inhibitor	6	ASA + any P2Y12 inhibitor	12	1000/1297	A composite of all-cause mortality, myocardial infarction, and cerebrovascular events)	—	BARC 2–5	—
EXCELLENT	ASA + clopidogrel	6	ASA + clopidogrel	12	744/1443	Composite of cardiac death, myocardial infarction, or target vessel revascularization	—	TIMI	—
IVUS XL	ASA + clopidogrel	6	ASA + clopidogrel	12	687/1400	Composite of cardiac death, myocardial infarction, stroke, or TIMI major bleeding	—	TIMI	—
OPTIMIZE	ASA + clopidogrel	3	ASA + clopidogrel	12	996/3119	Composite of death from any cause, MI, stroke, or major bleeding	—	Intracranial, intraocular, or retroperitoneal hemorrhage; clinically overt blood loss resulting hemoglobin decrease >3 g/dL, any hemoglobin decrease >4 g/dL, or transfusion of 1 unit of packed red blood cells or whole blood; or intracranial hemorrhage or bleeding causing hemodynamic compromise and requiring intervention	—
SECURITY	ASA + any P2Y12 inhibitor	6	ASA + any P2Y12 inhibitor	12	442/1399	Composite of cardiac death, MI, stroke, definite or probable stent thrombosis or bleeding academic consortium criteria (BARC) type 3 or 5 bleeding	—	BARC 3–5	—
NIPPON	ASA + clopidogrel (prasugrel)	6	ASA + clopidogrel (prasugrel)	18	1079/3307	All-cause death, Q-wave or non-Q-wave MI, cerebrovascular events, and major bleeding events	—	Modified REPLACE-2 criteria	—
STOPDAPT 2	ASA + clopidogrel	1	ASA + clopidogrel	12	1140/3009	Composite of cardiac death, MI, stroke, definite or probable stent thrombosis or TIMI major/minor bleeding	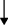	TIMI	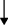
SMART CHOICE	ASA + any P2Y12 inhibitor	3	ASA + any P2Y12 inhibitor	12	1736/2993	All-cause death, MI, or stroke	—	BARC 2–5	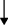
DES LATE-DATE	ASA + clopidogrel	12	ASA + clopidogrel	24	3063/5045	Composite of death resulting from cardiac causes, myocardial infarction, or stroke	—	TIMI	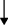
ARCTIC interruption	ASA + clopidogrel (75 or 150 mg) or prasugrel	12	ASA + clopidogrel (75 or 150 mg) or prasugrel	24	323/1286	Any death, myocardial infarction, stent thrombosis, stroke, or TIA, urgent revascularization	—	STEEPLE	—
DAPT study	ASA + clopidogrel/prasugrel	12	ASA + clopidogrel/prasugrel	30	3576/9961	Stent thrombosis	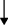	GUSTO	—
Italic/italic+	ASA	6	ASA + clopidogrel (prasugrel, ticagrelor)	12	792/1850	Composite of death, MI, emergency TVR, stroke, or major bleeding	—	TIMI	—
TWILIGHT	ASA + ticagrelor (then ticagrelor)	3	ASA + ticagrelor	12	7119/7119	Death from any cause, nonfatal myocardial infarction, or nonfatal stroke	—	BARC 2, 3–5	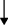
GLOBAL LEADERS	ASA + ticagrelor (then ticagrelor)	1	ASA + ticagrelor or clopidogrel	12	7487/15968	All-cause mortality or new nonfatal, centrally adjudicated Q-wave MI	—	BARC 3–5	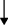

**Table 2:** Characteristics of randomized studies comparing different antiplatelet strategies in association with direct oral anticoagulants or vitamin K antagonists (VKA).

Study	Antiplatelet treatment				ACS patients (n/N)	Primary efficacy endpoint	Primary efficacy result	Primary safety endpoint	Primary safety result
	Strategy shorter DAPT	Months	Strategy longer DAPT	Months					
PIONEER AF-PCI	Rivaroxaban 15 + clopidogrel (ticagrelor/prasugrel allowed < 15%) or	12 months	VKA + DAPT	1, 6, or 12 months	1096/1415	Death from cardiovascular cause, myocardial infarction, or stroke	—	Clinically significant bleeding	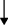
WOEST	Causes myocardial infarction or stroke						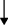	All bleeding events (TIMI criteria)	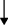
ENTRUST-AF PCI	VKA + clopidogrel	1 month after BMS and 12 months after DES	VKA + ASA + clopidogrel	1 month after BMS and 12 months after DES	155/563	Stroke, death, myocardial infarction, stent thrombosis, and target vessel revascularization	—	Clinically relevant bleeding	—
RE-DUAL PCI	Edoxaban 60 mg + clopidogrel ticagrelor/prasugrel allowed < 10%)	12 months	VKA + ASA + clopidogrel ticagrelor/prasugrel allowed < 15%)	12 months (ASA min 1 month)	777/1506	Cardiovascular death, stroke, systemic embolic events (SEE), myocardial infarction, and definite stent thrombosis	—	ISTH major or clinically relevant nonmajor bleedings	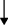
AUGUSTUS	Dabigatran 110 or 150 mg BID + clopidogrel or ticagrelor	6 months	VKA + ASA + clopidogrel or ticagrelor	6 months	1744/3489	Thromboembolic events, death, or unplanned revascularization	—	ISTH major or clinically relevant nonmajor bleedings	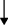
